# Analysis of Environmental Determinants of Heme and Nonheme Iron Intake in a National Sample of Polish Adolescents

**DOI:** 10.3390/ijerph18105252

**Published:** 2021-05-14

**Authors:** Dominika Skolmowska, Dominika Głąbska

**Affiliations:** Department of Dietetics, Institute of Human Nutrition Sciences, Warsaw University of Life Sciences (WULS-SGGW), 159C Nowoursynowska Street, 02-776 Warsaw, Poland; dominika_skolmowska@sggw.edu.pl

**Keywords:** adolescents, iron, sources, heme and nonheme iron, animal products, plant products, regions, Polish representative sample, anaemia

## Abstract

Intake of sufficient amounts of iron by adolescents is a matter of great concern. Therefore, it is crucial to determine the factors that may influence iron intake in this specific population. The present study aimed to analyze the environmental determinants of the intake of heme and nonheme iron in a national homogenous sample of Polish adolescents. Adolescents (aged 15–20 years) were randomly chosen from all the regions of Poland by performing a sampling of secondary schools (random quota sampling). The total iron intake, as well as the intake of heme iron, nonheme iron, animal iron, plant iron, and iron from various food products, was assessed among 1385 female respondents and 1025 male respondents using the validated IRON Intake Calculation—Food Frequency Questionnaire (IRONIC-FFQ). The intake was compared between the subgroups stratified by meat intake in the region, gross domestic product (GDP) in the region, and size of the city (rural vs. urban environment). It was observed that meat intake in the region did not influence the intake of total iron, as well as the intake of heme iron, nonheme iron, animal iron, plant iron, and iron from various food products (*p* > 0.05). However, GDP and the size of the city were determined as the most influencing factors, as they were associated with iron intake in both female and male adolescents, with the most prominent differences between the subgroups found in the case of females. Female adolescents from high-GDP regions had significantly higher intake of heme iron (*p* = 0.0047) and animal iron (*p* = 0.0029), and lower intake of nonheme iron compared to those from low-GDP regions (*p* = 0.0342). The total iron intake was higher among female adolescents who were from medium cities than those from big cities (*p* = 0.0350), but significantly higher animal iron intake (*p* = 0.0404) and plant iron intake (*p =* 0.0385) were observed among females from villages and small towns compared to females from other groups. Based on the results, it may be concluded that size of the city and the economic status of the region are the most important environmental determinants of iron intake in adolescents and, hence, they should be taken into account while developing educational programs, especially for the female adolescent population.

## 1. Introduction

Adolescence is a unique stage of life marked by biological, cognitive, and social changes [1]. During this period, health behaviors, including dietary choices, are most vulnerable [2] and may be influenced by both individual and environmental factors [3].

Environmental factors, in particular, may be decisive of food choices, as these factors affect not only the availability and choices of food products but also the development of specific preferences [4]. Environment can be distinguished into four different types as follows: physical, political, sociocultural, and economic [5]. However, all the factors associated with the environment may influence the availability of food products for purchase [6] and determine the food choices in a household [7].

Not only the choice of products but also the resultant nutritional value of the diet is governed by various factors, including the environmental factors [8]. However, the impact of the environment seems to be negligible for a number of nutrients [9]. The products that are most vulnerable to the effect of the indicated environmental factors include meat products [10], which are directly associated with iron intake and also show high variations depending on the environmental conditions [11]. 

One of the most important environmental factors is socioeconomic status. A study conducted by Clonan et al. [12] showed that this factor affects the intake of both red and processed meat. Consequently, the income of individuals may be perceived as one of the most important determinants of nutrient intake, as shown by the study of Knez et al. [13], in which no general differences in iron intake were observed, except for the differences between the low-income and affluent subgroups. 

Another important environmental determinant is the type of area, namely urban or rural environment, as studies have indicated some general differences in dietary habits and dietary intake based on this factor [14,15]. Various studies have shown that area influences the consumption of meat [16], as well as the resultant intake of iron [17] and other derived nutrients [18]. 

Taking into account the abovementioned differences, it can be stated that the environmental influence may be crucial, especially in the case of iron, for the prevention of diet-related diseases, the prevalence of which varies worldwide [19]. Such geographical disparity has been observed for anemia, as various proportions of the population are affected by this condition throughout the world. The global prevalence of anemia is estimated at 24.8%, but among young women, who are the most vulnerable group, its prevalence ranges from 17.8% to 47.5% [20]. The World Health Organization (WHO) has declared anemia as a general problem for public health with serious consequences [21]. Particularly in the case of children and adolescents, it is known that anemia adversely affects their behavioral characteristics, cognitive performance, and physical growth [22,23]. Therefore, providing adequate amounts of iron is important to meet individual iron requirements, and so it is crucial to determine the factors that may influence iron intake in specific populations. However, it should be noted that dietary iron is present in two chemical forms—as heme and nonheme—which differ in their bioavailability and uptake [24]. Heme iron, derived from animal products, is absorbed up to 25–35% [25], whereas nonheme iron, derived from both animal and plant products [26], is absorbed much less, in the range of 2–20% [27]. The greater efficiency of absorption of heme iron results from specific transporters that allow it to pass directly across cell membranes and into the bloodstream, while these transporters cannot be utilized by nonheme iron and require ferric iron (Fe^3+^) to be reduced to ferrous iron (Fe^2+^) prior to absorption [28]. 

Hence, understanding the determinants influencing iron intake may be of help to improve the dietary patterns of adolescents, as well as the resultant health status of this age group. This study was conducted with an aim of analyzing the environmental determinants of the intake of heme and nonheme iron in a national homogenous sample of Polish adolescents.

## 2. Materials and Methods

### 2.1. Ethical Statement

The study was conducted at the Department of Dietetics of the Warsaw University of Life Sciences (WULS-SGGW). The study was carried out in accordance with the guidelines of the Declaration of Helsinki. All the procedures were approved by the Ethics Committee of the Faculty of Human Nutrition and Consumer Sciences at the Warsaw University of Life Sciences (No 24/2018). Both the study participants and their parents/legal guardians submitted their written informed consent for participation in the study.

### 2.2. Studied Group

The study was carried out in a group of Polish secondary school students, aged 15–20 years. This age is typical for the secondary level of education in Poland, and the current net enrollment rate (NER) for secondary school students is 89.01% [29]. 

The participants were recruited during the period of November 2018 to February 2019 from all regions of Poland, based on geographical distribution. To gather a national sample of Polish adolescents, the sampling procedure was stratified and random quota sampling was performed with the quotas attributed to voivodeships and counties (voivodeships are the basic administrative units in Poland, which are comparable to provinces or states in other countries and are further divided into counties).

Sampling was conducted in two phases within this study: (1) in the main phase, 400 secondary schools were randomly selected; (2) in the subsidiary phase, 625 secondary schools were randomly selected. If the selected schools agreed to conduct the study, the further procedure was carried out according to the previously described methodology [30]. The procedure of inviting randomly chosen secondary schools is also detailed in the previous paper [30].

The inclusion criteria for students were as follows:

- Caucasian;
- Aged 15–20 years;
- Being a student of a randomly chosen secondary school;
- In the case of female respondents—declared regular menstrual cycle;
- Providing informed consent to take part in the study;
- In the case of minor participants—providing informed consent of parents/legal guardians to take part in the study.

The following students were excluded from the study:

- Female respondents who are currently pregnant/breastfeeding (due to their higher iron requirement compared to other women of childbearing age [17]);
- Those with iron intake higher than 45 mg (level of tolerable upper intake—UL)—interpreted as unreliable [31].

### 2.3. Applied Questionnaire

The study used an electronic questionnaire (IRON Intake Calculation—Food Frequency Questionnaire (IRONIC-FFQ)) which was forwarded to every student who agreed to participate in the study. The questionnaire has been validated and proven to provide reliable data in the Polish population [32] for the assessment of the intake of specific food products constituting iron sources. Iron intake was calculated using previously developed formulas [32], based on Polish food composition tables [33]. 

The iron intake was calculated as follows:

- Total iron intake, as well as the intake of various forms of iron—heme iron, nonheme iron, animal iron, and plant iron, as presented in the previous study [30], based on a commonly applied estimation [34];
- Intake of iron from the specific groups of products, as presented in the previous study [30].

The iron intake was compared separately for female and male respondents, between subgroups stratified by major environmental characteristics such as meat intake in the region, size of the city, and Gross Domestic Product (GDP) in the region. 

### 2.4. Statistical Analysis 

The sample size was calculated for the population of Polish adolescents aged 15–20 years (a total of 2,170,464, based on the data from the Central Statistical Office [35] in Poland), at a 95% confidence level and 5% margin of error. Assuming a percentage of 50%, which maximizes the sample size (due to the fact that no data on expected percentage of outcome were available), the required sample size was estimated at 384 respondents. Thus, the gathered sample of 2410 respondents was interpreted as sufficient.

The respondents were compared in subgroups, stratified based on the following characteristics:

- Meat intake in the region—this was estimated from the average meat intake in the voivodeship, and was categorized into groups of low and high meat intake, based on the calculations of the Polish Central Statistical Office [36]. Low meat intake in the region was defined as the intake of <5.08 kg per year (below-average intake for Poland) and high meat intake as the intake of ≥5.08 kg per year (above-average intake for Poland), as the average meat intake in Poland is 5.08 kg per year [36].
- Size of the city (rural/urban environment)—this was identified on the basis of the city size, and was categorized as villages and small towns (<20,000 inhabitants), medium cities, and big cities (>100,000 inhabitants) based on the calculations of the Polish Central Statistical Office [37].
- Socioeconomic status of the region—this was determined from the GDP for the voivodeship, based on the calculations of Eurostat [38], and was categorized into low GDP (24–54% in purchasing power standard), medium GDP, and high GDP (73–86% in purchasing power standard), as defined by Eurostat [38].

The statistical analysis was carried out using:

- Shapiro–Wilk test to verify normality of distribution;
- Chi^2^ test;
- Mann–Whitney U test;
- Kruskal–Wallis analysis of variance (ANOVA) with post hoc Tukey test;
- Multi-factor ANOVA in general linear model of three factors (meat intake in region, size of the city, and GDP for the voivodeship).

The statistical analysis was conducted using Statgraphics Plus for Windows 5.1 (Statgraphics Technologies Inc., The Plains, VA, USA).

## 3. Results

Table 1 presents intake of iron in the studied population-based sample of Polish female adolescents, stratified by meat intake in the region. There was no difference of intake of iron forms in the subgroups of female adolescents from the regions with low and high meat intake. At the same time, female adolescents from the regions with high meat intake were characterized by higher iron intake from fat, comparing to female adolescents from the regions with low meat intake (*p* = 0.0087), but not by higher iron intake from meat products (*p* = 0.8849).

Table 2 presents intake of iron in the studied population-based sample of Polish male adolescents, stratified by meat intake in the region. There was no difference of intake of iron forms, as well as in intake of iron from various food products, including meat products (*p* = 0.2937), in the subgroups of male adolescents from the regions with low and high meat intake. 

Table 3 presents intake of iron in the studied population-based sample of Polish female adolescents, stratified by GDP in the region. Female adolescents from the regions of high GDP were characterized by significantly higher heme iron (*p* = 0.0047) and animal iron (*p* = 0.0029) than those from the regions of low GDP. Female adolescents from the regions of low GDP were characterized by significantly higher nonheme iron intake than those of the regions with high GDP (*p* = 0.0342). At the same time, female adolescents from the regions of low GDP were characterized by significantly higher intake of iron from cereals and fruit than those from regions of low GDP (*p =* 0.0495; *p* = 0.0273, respectively). 

Table 4 presents intake of iron in the studied population-based sample of Polish male adolescents, stratified by GDP in the region. There was no difference of intake of iron forms. At the same time, male adolescents from the regions of low GDP were characterized by significantly higher iron intake from vegetables than those from the regions of medium GDP (*p* = 0.0180). Male adolescents from the regions of high GDP were characterized by significantly higher iron intake from cocoa than those from the regions of low and medium GDP (*p* = 0.0103). 

Table 5 presents intake of iron in the studied population-based sample of Polish female adolescents, stratified by the size of the city. Total iron intake was higher among female adolescents from medium cities than those from big cities (*p* = 0.0350). Female adolescents from villages and small towns were characterized by significantly higher animal iron intake (*p* = 0.0404) and plant iron intake (*p =* 0.0385) than those from big and medium cities, respectively. At the same time, female adolescents from villages and small towns were characterized by significantly higher iron intake from meat products (*p* = 0.0082), eggs (*p =* 0.0016), dairy products (*p* = 0.0034) and fish (*p* = 0.0010) than those from big cities. 

Table 6 presents intake of iron in the studied population-based sample of Polish male adolescents, stratified by the size of the city. There was no difference of intake of iron forms. At the same time, male adolescents from villages and small towns were characterized by significantly higher iron intake from nuts than those from medium and big cities (*p* = 0.0258). 

Table 7 presents the deepen analysis to test between-subjects effect in a general linear model of 3 factors (meat intake in region, size of the city, and GDP for the region). The conducted statistical analysis confirmed the previous observations formulated for a single factors analyzed. Namely, it was observed that the meat intake in the region was not an important factor influencing iron intake. At the same time, the presented analysis indicated that in the model including meat intake in region, environment, and GDP for the voivodeship, the size of the city was revealed to be significant determinant of iron intake in case of female adolescents.

## 4. Discussion

In the studied group, in the previous analysis [30], it was observed that male respondents were characterized by higher intake of various forms of iron compared to female respondents. This may be related to the fact that, compared to women, men consume a distinctly higher amount of meat, which is a source of highly absorbable heme iron [25,39]. Although women are more likely to follow a vegan or vegetarian diet [40], it should be indicated that independent of gender, the proportion of adolescents following vegetarian diets is increasing [41]. Moreover, the intake of most nutrients is correlated with energy intake [42] and, therefore, it may be assumed that in this study, male adolescents had higher energy intake and consequently higher iron intake than female adolescents. 

The study did not find any difference in iron intake, as well as in iron intake from various food products, in the subgroups stratified by meat intake in the region. Meat, particularly red meat, is rich in highly bioavailable heme iron [43], and thus, its adequate intake is necessary to prevent anemia [44], especially for women [45]. Studies show that a statistically significant difference in iron status can be observed between women of childbearing age who are following a diet that includes meat products, compared to those following a vegetarian diet [46]. On the other hand, high consumption of red meat is known to increase the risk of colorectal cancer [47]. Therefore, it is essential to maintain a proper balance between meat intake and the intake of other iron sources in order to prevent both anemia and colorectal cancer. In the present study, it was supposed that respondents from the regions with high habitual meat intake will be characterized by higher total iron intake and heme iron intake, but no differences were observed between this group and the other groups. Therefore, it can be considered that the other potential determinants of iron intake may also play an important role.

It was found that female adolescents from the regions of high GDP had significantly higher intake of heme iron and animal iron than those from low-GDP regions. However, no differences were noted in the total iron intake between the female adolescents from low- and high-GDP regions. This is due to the fact that the differences in the intake of heme iron were compensated by the differences in the intake of nonheme iron, as female respondents from the regions of low GDP were characterized by significantly higher intake of nonheme iron than those from the regions of high GDP. Nevertheless, it should be emphasized that heme iron is more effective than nonheme iron in preventing anemia due to its higher availability [48]. This corresponds with other results which suggest that socioeconomic status may affect iron intake [49,50]. In the study by Kim et al. [49], conducted in a group of Korean adolescent girls, it was observed that girls with a higher household income consumed more iron and had a lower prevalence of anemia, compared to those with a low income. Similarly, in the study by Akram et al. [50], carried out in a group of pregnant Pakistani women, women belonging to the upper class were characterized by higher iron intake than women belonging to the lower and middle class. Thus, it can be stated that inadequate iron supply may be a common problem in the low-economic-status group, but it may not be reflected only by total iron intake, and so, heme iron intake should also be monitored in this group. 

The type of residence (rural/urban environment) may influence the intake of some nutrients, including iron [51]. Urban areas are associated with higher energy density and higher consumption of purchased goods [52]. Moreover, rural settings are known to have a higher prevalence of anemia [53] and stunting [54], compared to urban settings. However, studies show that iron intake in rural areas is not actually lower than in urban areas, but is higher indeed [51,55,56]. For instance, the study by Martin et al. [51] reported that Australian women of reproductive age from rural areas had higher intake of iron than urban women. Similarly, the Bosnian study by Alibabić et al. [56] showed that rural women were characterized by significantly higher iron intake than their urban counterparts. Similar results regarding the influence of the type of residence on iron intake were obtained in the present study, as the total iron intake was found to be significantly higher among adolescent girls from medium cities than those from big cities. Additionally, adolescent girls from villages and small towns had significantly higher animal iron and plant iron intake compared to girls from medium and big cities At the same time, these female adolescents were characterized by significantly higher iron intake from certain products, such as meat, eggs, and dairy products, than those from big cities. Such results may be, to some extent, explained by various dietary patterns which are, in general, observed in rural and urban areas, as it is well known that the environment may influence the diet [15]. Urbanization may induce people to adopt a Western diet [57] which is characterized by a high proportion of energy-dense and processed foods and [58] and a low proportion of fruit and vegetables [59]. Therefore, it can be assumed that the lower iron intake observed in respondents from big cities in the present study might be because their diet is unbalanced and lacks essential nutrients, such as iron. 

Anemia is an important health problem in both developing and developed countries [60]. It is associated with the impairment of oxygen transport [61] and affects the physical and mental well-being of an individual [62]. One of the Global Nutrition Targets 2025 set by the WHO is a 50% reduction in the prevalence of anemia among women of childbearing age by the year 2025 [63]. Therefore, understanding the possible determinants of iron intake may be a key factor in establishing effective public health strategies aimed at preventing and controlling anemia. According to the WHO recommendations, such strategies should focus on improving dietary diversity and promoting the use of iron-fortified foods as well as iron supplements [63]. While iron fortification of staple food products is recommended in developing countries, in Poland it is not common, as mostly cereals and corn flakes are iron-fortified [64,65]. Moreover, it seems that communication campaigns may help in decreasing the prevalence of anemia among women and children [66]. However, no such educational campaign aiming at the prevention of anemia has been conducted in Poland so far. It is also indicated that nutritional education strategies achieve limited success when implemented alone; therefore, they should be applied along with fortification or supplementation programs [67]. Understanding the factors that influence dietary iron intake, such as the socioeconomic status of the region or the size of the city (rural/urban environment), will help to create cost-effective strategies targeted at specific population groups in the regions affected by anemia and insufficient iron intake. 

Although the study was conducted in a large national homogenous group of Polish adolescents and interesting results were obtained, there are some limitations to be indicated. The most important one is that the study was conducted only in a population of Polish adolescents, and so it does not provide a broader international perspective, which would be valuable. Another limitation is the fact that iron intake was assessed without the general energy value of diet, so it was impossible to recalculate iron per energy value of diet to compare not only intake of iron but also iron density of the diet. Last but not least, the data on the potential interfering factors were not gathered within the study (e.g., menstruation age, parents’ education), so they should be taken into account in further studies.

## 5. Conclusions

Based on the results obtained, it may be concluded that the economic status of the region and size of the city are the most important environmental determinants of iron intake in adolescents and, hence, they should be taken into account while developing educational programs, especially for the female adolescent population.

## Figures and Tables

**Table 1:** Intake of iron in a population-based sample of Polish female adolescents, stratified by meat intake in the region.

Iron Intake		Low Meat Intake in the Region				High Meat Intake in the Region				*p-*Value **
		Mean ± SD	Median	Min	Max	Mean ± SD	Median	Min	Max	
Intake of various forms of iron	Total iron (mg)	12.77 ± 6.99	11.01 *	0.48	43.59	12.83 ± 7.23	11.01 *	1.94	44.88	0.8445
	Heme iron (mg)	1.70 ± 1.55	1.16 *	0.00	9.86	1.67 ± 1.52	1.18 *	0.00	11.38	0.9534
	Nonheme iron (mg)	11.07 ± 5.96	9.72 *	0.38	40.84	11.16 ± 6.25	9.61 *	1.70	43.73	0.7876
	Animal iron (mg)	4.24 ± 3.87	2.91 *	0.00	24.65	4.18 ± 3.79	2.95 *	0.00	28.45	0.9532
	Plant iron (mg)	8.53 ± 4.95	7.56 *	0.00	7.84	8.65 ± 5.29	7.34 *	0.00	42.00	0.7901
Intake of iron from various food products	Cereals (mg)	3.59 ± 2.29	3.15 *	0.00	17.34	3.57 ± 2.30	3.04 *	0.00	19.93	0.5817
	Meat products (mg)	3.34 ± 3.66	2.02 *	0.00	24.21	3.27 ± 3.56	2.04 *	0.00	24.46	0.8849
	Vegetables (mg)	2.21 ± 2.02	1.60 *	0.00	12.07	2.24 ± 2.12	1.60 *	0.00	14.43	0.8537
	Nuts (mg)	0.97 ± 1.26	0.64 *	0.00	10.84	1.09 ± 1.52	0.72 *	0.00	20.24	0.3785
	Fruit (mg)	0.74 ± 0.66	0.56 *	0.00	6.44	0.70 ± 0.63	0.55 *	0.00	7.34	0.3012
	Cocoa (mg)	0.53 ± 0.57	0.36 *	0.00	4.34	0.53 ± 0.57	0.36 *	0.00	4.71	0.8447
	Eggs (mg)	0.52 ± 0.56	0.47 *	0.00	6.29	0.53 ± 0.44	0.47 *	0.00	3.14	0.1574
	Potatoes (mg)	0.36 ± 0.32	0.29 *	0.00	3.57	0.38 ± 0.38	0.29 *	0.00	3.57	0.2694
	Dairy products (mg)	0.27 ± 0.20	0.24 *	0.00	1.81	0.27 ± 0.18	0.24 *	0.00	1.87	0.3037
	Fat (mg)	0.13 ± 0.12	0.09 *	0.00	1.43	0.14 ± 0.15	0.11 *	0.00	1.71	0.0087
	Fish products (mg)	0.11 ± 0.16	0.06 *	0.00	1.38	0.11 ± 0.16	0.06 *	0.00	1.71	0.7239

* nonparametric distribution (for Shapiro–Wilk test *p* ≤ 0.05). ** Mann–Whitney U test used (nonparametric distribution).

**Table 2:** Intake of iron in a population-based sample of Polish male adolescents, stratified by meat intake in the region.

Iron Intake		Low Meat Intake in the Region				High Meat Intake in the Region				*p-*Value **
		Mean ± SD	Median	Min	Max	Mean ± SD	Median	Min	Max	
Intake of various forms of iron	Total iron (mg)	17.56 ± 9.16	15.50 *	2.06	44.74	18.11 ± 9.42	15.81 *	3.44	43.53	0.5202
	Heme iron (mg)	3.01 ± 2.16	2.43 *	0.00	13.08	3.12 ± 2.20	2.66 *	0.18	13.62	0.4365
	Nonheme iron (mg)	14.55 ± 7.62	12.73 *	1.25	39.81	14.99 ± 7.80	12.85 *	2.65	37.12	0.5593
	Animal iron (mg)	7.53 ± 5.39	6.06 *	0.00	32.70	7.80 ± 5.51	6.64 *	0.44	34.04	0.4365
	Plant iron (mg)	10.03 ± 6.06	8.75 *	0.00	19.59	10.31 ± 6.12	8.56 *	1.47	20.26	0.6747
Intake of iron from various food products	Cereals (mg)	4.50 ± 3.21	3.78 *	0.00	26.53	4.54 ± 3.10	3.80 *	0.00	15.96	0.7894
	Meat products (mg)	6.08 ± 5.00	4.60 *	0.00	31.25	6.41 ± 5.13	5.03 *	0.00	31.81	0.2937
	Vegetables (mg)	2.46 ± 2.38	1.76 *	0.00	15.21	8.61 ± 2.40	1.94 *	0.00	11.91	0.7787
	Nuts (mg)	1.06 ± 1.56	0.72 *	0.00	14.61	1.02 ± 1.54	0.55 *	0.00	14.05	0.3934
	Fruit (mg)	0.70 ± 0.68	0.46 *	0.00	6.47	0.73 ± 0.69	0.46 *	0.00	4.61	0.8951
	Cocoa (mg)	0.60 ± 0.66	0.42 *	0.00	4.81	0.70 ± 0.81	0.48 *	0.00	5.72	0.3368
	Eggs (mg)	0.90 ± 0.93	0.63 *	0.00	6.29	0.83 ± 0.93	0.47 *	0.00	6.29	0.0580
	Potatoes (mg)	0.54 ± 0.57	0.36 *	0.00	3.57	0.61 ± 0.74	0.36 *	0.00	3.57	0.6951
	Dairy products (mg)	0.36 ± 0.28	0.28 *	0.00	2.04	0.33 ± 0.26	0.26 *	0.00	1.64	0.1183
	Fat (mg)	0.18 ± 0.22	0.11 *	0.00	1.97	0.17 ± 0.22	0.09 *	0.00	1.43	0.1339
	Fish products (mg)	0.19 ± 0.25	0.13 *	0.00	2.03	0.24 ± 0.34	0.13 *	0.00	1.96	0.2344

* nonparametric distribution (for Shapiro–Wilk test *p* ≤ 0.05). ** Mann–Whitney U test used (nonparametric distribution).

**Table 3:** Intake of iron in a population-based sample of Polish female adolescents, stratified by Gross Domestic Product (GDP) in the region.

Iron Intake		Low GDP in the Region				Medium GDP in the Region				High GDP in the Region				*p*-Value **
		Mean ± SD	Median	Min	Max	Mean ± SD	Median	Min	Max	Mean ± SD	Median	Min	Max	
Intake of various forms of iron	Total iron (mg)	13.03 ± 7.44	11.06 *	1.58	43.59	12.71 ± 6.88	11.04 *	0.48	44.88	12.43 ± 6.83	10.80 *	1.74	37.17	0.4556
	Heme iron (mg)	1.66 ± 1.52	1.14 *^A^	0.00	10.32	1.68 ± 1.47	1.20 *^A^	0.00	11.38	1.76 ± 1.72	1.17 *^B^	0.03	8.39	0.0047
	Nonheme iron (mg)	11.38 ± 6.49	9.78 *^A^	1.58	40.84	11.03 ± 5.92	9.72 *^AB^	0.38	43.73	10.67 ± 5.52	9.39 *^B^	1.55	29.12	0.0342
	Animal iron (mg)	4.14 ± 3.81	2.86 *^A^	0.00	25.81	4.21 ± 3.67	3.01 *^A^	0.00	28.45	4.41 ± 4.30	2.92 *^B^	0.07	20.97	0.0029
	Plant iron (mg)	8.89 ± 5.57	7.40 *	0.00	20.61	8.50 ± 4.97	7.59 *	0.23	42.00	8.02 ± 4.17	7.10 *	1.18	22.72	0.6638
Intake of iron from various food products	Cereals (mg)	3.66 ± 2.41	3.08 *^A^	0.00	19.93	3.53 ± 2.18	3.07 *^AB^	0.05	16.91	3.49 ± 2.28	3.04 *^B^	0.26	16.40	0.0495
	Meat products (mg)	3.22 ± 3.57	1.93 *	0.00	22.69	3.28 ± 3.47	2.05 *	0.00	24.46	3.60 ± 4.04	2.22 *	0.00	20.47	0.2370
	Vegetables (mg)	2.37 ± 2.25	1.60 *	0.00	12.86	2.16 ± 1.97	1.60 *	0.00	14.43	2.01 ± 1.77	1.44 *	0.00	11.66	0.1608
	Nuts (mg)	1.07 ± 1.31	0.72 *	0.00	10.84	1.05 ± 1.56	0.55 *	0.00	20.24	0.86 ± 1.15	0.37 *	0.00	7.23	0.5640
	Fruit (mg)	0.76 ± 0.67	0.64 *^A^	0.00	6.44	0.71 ± 0.66	0.55 *^AB^	0.00	7.34	0.63 ± 0.52	0.55 *^B^	0.00	4.14	0.0273
	Cocoa (mg)	0.53 ± 0.55	0.36 *	0.00	3.76	0.52 ± 0.58	0.36 *	0.00	4.71	0.52 ± 0.59	0.41 *	0.00	3.87	0.9303
	Eggs (mg)	0.53 ± 0.48	0.47 *	0.00	6.29	0.53 ± 0.48	0.47 *	0.00	4.71	0.47 ± 0.58	0.31 *	0.00	6.29	0.7161
	Potatoes (mg)	0.36 ± 0.35	0.29 *	0.00	3.57	0.38 ± 0.38	0.29 *	0.00	3.57	0.39 ± 0.32	0.29 *	0.00	2.86	0.5341
	Dairy products (mg)	0.28 ± 0.20	0.24 *	0.00	1.87	0.28 ± 0.19	0.25 *	0.00	1.79	0.24 ± 0.19	0.21 *	0.00	1.32	0.5437
	Fat (mg)	0.14 ± 0.12	0.11 *	0.00	1.14	0.14 ± 0.16	0.11 *	0.00	1.71	0.12 ± 0.10	0.11 *	0.00	0.80	0.5341
	Fish products (mg)	0.11 ± 0.14	0.06 *	0.00	1.71	0.12 ± 0.18	0.06 *	0.00	1.38	0.10 ± 0.14	0.06 *	0.00	1.02	0.7093

* nonparametric distribution (for Shapiro–Wilk test *p* ≤ 0.05). ** Kruskal–Wallis analysis of variance (ANOVA) with post hoc Tukey test used (nonparametric distribution)–values with different letters in rows (A, B) are significantly different.

**Table 4:** Intake of iron in a population-based sample of Polish male adolescents, stratified by Gross Domestic Product (GDP) in the region.

Iron intake		Low GDP in the Region				Medium GDP in the Region				High GDP in the Region				*p*-Value **
		Mean ± SD	Median	Min	Max	Mean ± SD	Median	Min	Max	Mean ± SD	Median	Min	Max	
Intake of various forms of iron	Total iron (mg)	18.59 ± 9.55	16.52 *	2.95	43.62	17.30 ± 9.05	15.26 *	2.06	44.74	20.58 ± 10.28	20.70	5.82	33.04	0.5323
	Heme iron (mg)	3.25 ± 2.31	2.64 *	0.18	13.62	2.95 ± 2.11	2.37 *	0.00	13.08	3.11 ± 1.96	3.27 *	0.59	5.35	0.1193
	Nonheme iron (mg)	15.34 ± 7.89	13.53 *	2.65	37.12	14.35 ± 7.54	12.53 *	1.25	39.81	17.47 ± 8.98	15.64	4.91	29.76	0.4329
	Animal iron (mg)	8.12 ± 5.78	6.59 *	0.44	34.04	7.38 ± 5.28	5.92 *	0.00	32.70	7.78 ± 4.91	8.19	1.49	13.37	0.2324
	Plant iron (mg)	10.47 ± 6.24	9.20 *	1.47	10.91	9.92 ± 5.99	8.57 *	0.00	37.61	12.80 ± 7.59	10.53 *	3.54	24.85	0.7868
Intake of iron from various food products	Cereals (mg)	4.52 ± 3.17	3.89 *	0.00	22.56	4.49 ± 3.19	3.78 *	0.00	26.53	5.39 ± 4.26	4.25 *	1.59	14.54	0.0656
	Meat products (mg)	6.46 ± 5.43	4.82 *	0.00	31.81	6.03 ± 4.88	4.73 *	0.00	30.61	6.01 ± 4.59	4.18 *	0.67	11.73	0.1557
	Vegetables (mg)	2.68 ± 2.42	2.10 *^A^	0.00	11.29	2.40 ± 2.36	1.60 *^B^	0.00	15.21	3.03 ± 3.48	2.41 *^AB^	0.00	10.31	0.0180
	Nuts (mg)	1.19 ± 1.82	0.72 *	0.00	14.05	1.01 ± 1.45	0.72 *	0.00	14.61	0.67 ± 0.76	0.37 *	0.00	2.17	0.0563
	Fruit (mg)	0.80 ± 0.79	0.55 *	0.00	5.01	0.66 ± 0.61	0.46 *	0.00	5.54	1.50 ± 2.22	0.83	0.19	6.47	0.3031
	Cocoa (mg)	0.60 ± 0.72	0.39 *^A^	0.00	4.44	0.62 ± 0.68	0.42 *^A^	0.00	5.72	1.27 ± 0.87	1.00^B^	0.45	2.99	0.0103
	Eggs (mg)	1.06 ± 1.15	0.63 *	0.00	6.29	0.82 ± 0.82	0.63 *	0.00	6.29	1.10 ± 1.29	0.63	0.31	3.93	0.0952
	Potatoes (mg)	0.50 ± 0.59	0.36 *	0.00	3.57	0.57 ± 0.61	0.36 *	0.00	3.57	0.60 ± 0.69	0.36	0.21	2.14	0.1285
	Dairy products (mg)	0.37 ± 0.29	0.28 *	0.00	1.73	0.34 ± 0.27	0.28 *	0.00	2.04	0.46 ± 0.31	0.37	0.05	0.90	0.2975
	Fat (mg)	0.17 ± 0.25	0.09 *	0.00	1.43	0.17 ± 0.21	0.11 *	0.00	1.97	0.33 ± 0.38	0.14	0.09	1.14	0.1285
	Fish products (mg)	0.22 ± 0.31	0.13 *	0.00	1.96	0.19 ± 0.26	0.13 *	0.00	2.03	0.21 ± 0.26	0.13	0.00	0.72	0.0988

* nonparametric distribution (for Shapiro–Wilk test *p* ≤ 0.05). ** Kruskal–Wallis analysis of variance (ANOVA) with post hoc Tukey test used (nonparametric distribution)–values with different letters in rows (A, B) are significantly different.

**Table 5:** Intake of iron in a population-based sample of Polish female adolescents, stratified by the size of the city.

Iron Intake		Villages and Small Towns				Medium Cities				Big Cities				*p*-Value **
		Mean ± SD	Median	Min	Max	Mean ± SD	Median	Min	Max	Mean ± SD	Median	Min	Max	
Intake of various forms of iron	Total iron (mg)	13.35 ± 7.25	11.42 *^AB^	1.58	43.59	12.46 ± 6.97	10.99 *^A^	0.48	44.88	11.16 ± 6.73	9.72 *^B^	2.22	38.23	0.0350
	Heme iron (mg)	1.73 ± 1.61	1.15 *	0.00	11.38	1.68 ± 1.46	1.22 *	0.00	9.78	1.39 ± 1.46	1.01 *	0.01	9.86	0.0527
	Nonheme iron (mg)	11.62 ± 6.25	10.23 *	1.55	40.84	10.79 ± 5.96	9.40 *	0.38	43.73	9.77 ± 5.80	8.62 *	2.19	33.53	0.8179
	Animal iron (mg)	4.33 ± 4.02	2.87 *^A^	0.00	28.45	4.19 ± 3.64	3.05 *^AB^	0.00	24.44	3.47 ± 3.64	2.52 *^B^	0.02	24.65	0.0404
	Plant iron (mg)	9.02 ± 5.34	7.79 *^A^	0.00	7.84	8.27 ± 4.89	7.13 *^B^	0.23	42.00	7.69 ± 4.93	6.52 *^AB^	1.52	33.19	0.0385
Intake of iron from various food products	Cereals (mg)	3.71 ± 2.33	3.24 *	0.00	17.34	3.48 ± 2.23	2.92 *	0.00	16.91	3.29 ± 2.44	3.00 *	0.26	19.93	0.1293
	Meat products (mg)	3.40 ± 3.76	1.94 *^A^	0.00	24.46	3.30 ± 3.45	2.16 *^AB^	0.00	22.69	2.63 ± 3.53	1.53 *^B^	0.00	24.21	0.0082
	Vegetables (mg)	2.39 ± 2.21	1.76 *	0.00	14.43	2.12 ± 1.95	1.57 *	0.00	12.86	1.75 ± 1.62	1.29 *	0.00	10.16	0.7926
	Nuts (mg)	1.09 ± 1.40	0.72 *	0.00	10.84	0.99 ± 1.41	0.55 *	0.00	20.24	0.92 ± 1.38	0.37 *	0.00	7.23	0.9542
	Fruit (mg)	0.78 ± 0.74	0.64 *	0.00	7.34	0.67 ± 0.54	0.55 *	0.00	5.56	0.71 ± 0.63	0.55 *	0.00	3.69	0.1472
	Cocoa (mg)	0.55 ± 0.55	0.42 *	0.00	4.71	0.51 ± 0.59	0.36 *	0.00	4.34	0.46 ± 0.53	0.33 *	0.00	4.23	0.1153
	Eggs (mg)	0.54 ± 0.49	0.47 *^A^	0.00	6.29	0.52 ± 0.47	0.47 *^AB^	0.00	4.71	0.48 ± 0.71	0.31 *^B^	0.00	6.29	0.0016
	Potatoes (mg)	0.37 ± 0.34	0.29 *	0.00	3.57	0.37 ± 0.36	0.29 *	0.00	3.57	0.41 ± 0.46	0.36 *	0.00	3.57	0.0541
	Dairy products (mg)	0.28 ± 0.20	0.25 *^A^	0.00	1.81	0.26 ± 0.18	0.23 *^B^	0.00	1.87	0.25 ± 0.23	0.21 *^B^	0.00	1.79	0.0034
	Fat (mg)	0.14 ± 0.14	0.11 *	0.00	1.71	0.14 ± 0.13	0.11 *	0.00	1.43	0.14 ± 0.14	0.11 *	0.00	0.86	0.0541
	Fish products (mg)	0.11 ± 0.16	0.06 *^A^	0.00	1.71	0.11 ± 0.16	0.06 *^B^	0.00	1.25	0.10 ± 0.12	0.06 *^B^	0.00	0.59	0.0010

* nonparametric distribution (for Shapiro–Wilk test *p* ≤ 0.05). ** Kruskal–Wallis analysis of variance (ANOVA) with post hoc Tukey test used (nonparametric distribution)–values with different letters in rows (A, B) are significantly different.

**Table 6:** Intake of iron in a population-based sample of Polish male adolescents, stratified by the size of the city.

Iron Intake		Villages and Small Towns				Medium Cities				Big Cities				*p*-Value **
		Mean ± SD	Median	Min	Max	Mean ± SD	Median	Min	Max	Mean ± SD	Median	Min	Max	
Intake of various forms of iron	Total iron (mg)	17.97 ± 9.30	16.03 *	2.67	44.74	17.49 ± 9.17	15.29 *	2.09	43.62	15.55 ± 8.22	13.95 *	2.06	37.17	0.2751
	Heme iron (mg)	3.07 ± 2.14	2.48 *	0.20	13.62	3.01 ± 2.20	2.37 *	0.00	13.08	2.69 ± 2.13	2.32 *	0.00	11.34	0.9783
	Nonheme iron (mg)	14.90 ± 7.75	12.94 *	2.27	39.81	14.47 ± 7.59	12.50 *	1.25	37.61	12.86 ± 6.86	11.53 *	1.50	33.95	0.1406
	Animal iron (mg)	7.68 ± 5.35	6.20 *	0.49	34.04	7.53 ± 5.51	5.93 *	0.00	32.70	6.74 ± 5.32	5.80 *	0.00	28.35	0.3230
	Plant iron (mg)	10.30 ± 6.13	8.95 *	1.47	8.92	9.95 ± 6.01	8.57 *	0.00	37.61	8.81 ± 5.76	7.69 *	0.66	29.12	0.2478
Intake of iron from various food products	Cereals (mg)	4.53 ± 3.19	3.86 *	0.00	26.53	4.55 ± 3.20	3.76 *	0.00	18.09	3.84 ± 3.05	2.85 *	0.26	15.29	0.3982
	Meat products (mg)	6.22 ± 4.96	4.82 *	0.00	31.81	6.14 ± 5.16	4.56 *	0.00	31.25	5.27 ± 4.45	3.84 *	0.00	18.97	0.5999
	Vegetables (mg)	2.52 ± 2.36	1.94 *	0.00	14.51	2.43 ± 2.43	1.60 *	0.00	15.21	2.43 ± 2.22	2.26 *	0.00	11.29	0.8223
	Nuts (mg)	1.16 ± 1.79	0.72 *^A^	0.00	14.61	0.95 ± 1.27	0.55 *^B^	0.00	12.25	0.71 ± 0.83	0.45 *^AB^	0.00	3.61	0.0258
	Fruit (mg)	0.74 ± 0.74	0.55 *	0.00	6.47	0.67 ± 0.64	0.46 *	0.00	5.54	0.58 ± 0.41	0.46 *	0.09	2.04	0.0668
	Cocoa (mg)	0.62 ± 0.73	0.42 *	0.00	5.72	0.63 ± 0.66	0.45 *	0.00	4.81	0.50 ± 0.54	0.43 *	0.00	3.17	0.3588
	Eggs (mg)	0.90 ± 0.97	0.63 *	0.00	6.29	0.87 ± 0.85	0.63 *	0.00	6.29	0.94 ± 1.14	0.63 *	0.00	6.29	0.2050
	Potatoes (mg)	0.55 ± 0.59	0.36 *	0.00	3.57	0.55 ± 0.61	0.36 *	0.00	3.57	0.59 ± 0.77	0.36 *	0.00	3.57	0.3218
	Dairy products (mg)	0.35 ± 0.29	0.27 *	0.00	1.73	0.35 ± 0.25	0.29 *	0.00	2.04	0.35 ± 0.37	0.26 *	0.00	2.00	0.1877
	Fat (mg)	0.17 ± 0.22	0.11 *	0.00	1.43	0.18 ± 0.23	0.11 *	0.00	1.97	0.17 ± 0.13	0.14 *	0.00	0.57	0.3217
	Fish products (mg)	0.21 ± 0.28	0.13 *	0.00	1.96	0.18 ± 0.26	0.06 *	0.00	2.03	0.18 ± 0.30	0.10 *	0.00	1.50	0.2074

* nonparametric distribution (for Shapiro–Wilk test *p* ≤ 0.05). ** Kruskal–Wallis analysis of variance (ANOVA) with post hoc Tukey test used (nonparametric distribution)–values with different letters in rows (A, B) are significantly different.

**Table 7:** Analysis to test between-subjects effect in a general linear model of 3 factors (meat intake in region, size of the city, and GDP for the region) (multi-factor ANOVA**–**p-Values presented).

Iron Intake	Female Adolescents			Male Adolescents		
	Meat Intake in the Region	GDP in the Region	Size of the City	Meat Intake in the Region	GDP in the Region	Size of the City
Total iron	0.9331	0.8768	0.0101	0.5220	0.1110	0.2134
Heme iron	0.9019	0.4947	0.0872	0.5735	0.1501	0.4322
Nonheme iron	0.9467	0.6708	0.0096	0.5413	0.1321	0.2133
Animal iron	0.9019	0.4947	0.0872	0.5735	0.1501	0.4322
Plant iron	0.9806	0.2792	0.0178	0.6396	0.2561	0.2931

GDP—Gross Domestic Product.

## Data Availability

No new data were created or analyzed in this study. Data sharing is not applicable to this article.
